# Whole Genome Resequencing of *Capsicum baccatum* and *Capsicum annuum* to Discover Single Nucleotide Polymorphism Related to Powdery Mildew Resistance

**DOI:** 10.1038/s41598-018-23279-5

**Published:** 2018-03-26

**Authors:** Yul-Kyun Ahn, Abinaya Manivannan, Sandeep Karna, Tae-Hwan Jun, Eun-Young Yang, Sena Choi, Jin-Hee Kim, Do-Sun Kim, Eun-Su Lee

**Affiliations:** 1 Department of Vegetable Crops, Korea National College of Agriculture and Fisheries, Jeonju, 54874 Republic of Korea; 2 Vegetable Research Division, National Institute of Horticultural and Herbal Science, Rural Development Administration, Jeonju, 55365 Republic of Korea; 3 Department of Plant Bioscience, Pusan National University, Busan, 46241 Republic of Korea

## Abstract

The present study deals with genome wide identification of single-nucleotide polymorphism (SNP) markers related to powdery mildew (PM) resistance in two pepper varieties. *Capsicum baccatum* (PRH1- a PM resistant line) and *Capsicum annuum* (Saengryeg- a PM susceptible line), were resequenced to develop SNP markers. A total of 6,213,009 and 6,840,889 SNPs for PRH1 and Saengryeg respectively have been discovered. Among the SNPs, majority were classified as homozygous type SNPs, particularly in the resistant line. Moreover, the SNPs were differentially distributed among the chromosomes in both the resistant and susceptible lines. In total, 4,887,031 polymorphic SNP loci were identified between the two lines and 306,871 high-resolution melting (HRM) marker primer sets were designed. In order to understand the SNPs associated with the vital genes involved in diseases resistance and stress associated processes, chromosome-wise gene ontology analysis was performed. The results revealed the occurrence that SNPs related to diseases resistance genes were predominantly distributed in chromosome 4. In addition, 6281 SNPs associated with 46 resistance genes were identified. Among the lines, PRH1 consisted of maximum number of polymorphic SNPs related to NBS-LRR genes. The SNP markers were validated using HRM assay in 45 F_4_ populations and correlated with the phenotypic disease index.

## Introduction

Chili pepper is an economically important horticultural crop in Solanaceae family that also includes potato, tomato, eggplant, petunia and tobacco. The Solanaceae family includes more than 3,000 varied species with the similar numbers of chromosomes (n = 12) but significantly different genomic sizes. Peppers have been used as a vegetable, condiment, spice, medicine, coloring agent and source of vitamins^1–3^. The most common cultivated pepper species are *Capsicum annuum*, *Capsicum frutescens*, *Capsicum chinense*, *Capsicum pubescens*, and *Capsicum baccatum*^4,5^. Though pepper consists of several potential economic values, fungi, bacteria and viruses cause heavy losses in pepper fruit production. Powdery mildew (PM) is the most common devastating fungal disease in pepper and is caused by *Leveillulataurici*. In an agricultural setting, this disease could be controlled using agrochemicals or genetic resistance lines. The selection of good PM resistance varieties through traditional breeding potentially requires more than 10 years. Hence, molecular marker-assisted breeding is the current plant breeding method of choice, and the most frequently used markers include single-nucleotide polymorphisms (SNPs). DNA-based molecular markers are employed in plant breeding for genetic diversity and genome association analyses^6–9^. Over the last decade, major innovations in sequencing technologies and bioinformatics have been achieved, prompting a transition from classical conservation genetics to conservation genomics^10–13^. Rapid innovations in genome sequencing platforms, such as next generation sequencing (NGS), provide numerous opportunities for transcriptome assembly, functional annotation of genes, and identification of molecular markers^14,15^. New software tools in NGS technology enable the cost effective identification, confirmation, and evaluation of genetic markers on a large scale.

SNPs have been accepted as potential selection markers in genome-wide studies given the high density of markers near loci of interest^6^. NGS technologies have identified genome-wide SNPs in several crops, such as bean^16^, barley^17^, cassava^18^, cabbage^19^, grape^20^ and maize^21^. In pepper, several thousand genetic markers, especially SNPs have been discovered^22–28^. Recently, Kim *et al*.^29^ sequenced and assembled the pepper genome (*Capsicum annuum cv*. CM334) at a genomic size of 3.48 Gb. This reference genome will provide the opportunity to improve quality, cultivation, and disease resistance in *Capsicum* species. The aim of this research is to discover SNP variants for future marker-assisted breeding studies related to PM resistance using *Capsicum annuum cv*. CM334 as a reference for data mining. Thus, in the present study resequencing of two pepper varieties, *Capsicum baccatum* (PRH1- PM resistant line) and *Capsicum annuum* (Saengryeg - PM susceptible line), using the HiSeq. 4000 Illumina platform and the genome wide identification of SNPs have been implemented

## Results

### Genome sequencing, pre-processing and alignment of reads to the reference genome

A summary of the sequencing, sequence preprocessing, and alignment to the read mapping were presented in Table 1. In total, 130,370,103 and 118,588,231 paired-raw reads were discovered for PRH1 and Saengryeg, respectively, with an average length of 151 bp. A total of 19.69 and 17.91 Gb paired-end raw reads were recorded for both pepper varieties. The total genome coverages were ≒ 11.31× and ≒ 10.29× of the reference genome. The Solexa QA (v.1.13) package was used to generate high-quality clean reads. Raw reads were assessed for quality, and impractical parts were discarded. After the removal of adaptor sequences, ambiguous and low-quality reads (Q value <20), a total of 97,216,537 and 88,964,871 reads were discovered for PM resistant and susceptible pepper varieties, with ≒ 5.61× and ≒ 5.17× of genome coverage respectively. After the removal of non-specific reads, the remaining reads were mapped to the reference genome. A total of 194,523,074 and 177,929,742 clean, high-quality reads were recorded for PRH1 and Saengryeg, respectively, compared with the reference genome, covering 88,448,386 (45.47%) and 1,080,500,795 (39.24%) of mapped reads, respectively.

**Table 1:** Summary of sequencing, sequence pre-processing and alignment of reads to the reference genome.

Sample	Read parameters	PRH1	Saengryeg
Raw read data	No. of reads	130,370,103	118,588,231
		130,370,103	118,588,231
	Avg. length (bp)	151	151
		151	151
	Total length (Gb)	19.69	17.91
		19.69	17.91
	Genome coverage#	≒11.31X	≒10.29X
Cleaned data	No. of reads	97,261,537	88,964,871
		97,261,537	88,964,871
	Avg. length (bp)	120	121
		81	81
	Total length (Gb)	11.69	10.79
		7.83	7.21
	Trimmed/raw*	59.41	60.24
		39.78	40.25
	Genome coverage#	≒5.61×	≒5.71X
Read mapping	No. of total reads	194,523,074	177,929,742
	No. of mapped reads (%)	88,448,386 (45.47)	1,080,500,795 (39.24)
	Mapped region** (%)	1,080,500,765 (39.24)	2,514,912,154 (91.34)

^*^Trimmed/raw: total length of trimmed read / total length of raw read.

^#^Genome coverage: Total length of all reads divided by reference genome size (3.48 Gb).

^**^Mapped region: Coverage of read mapping relative to the reference genome.

### Identification and distribution of SNP markers

**Figure 1:**
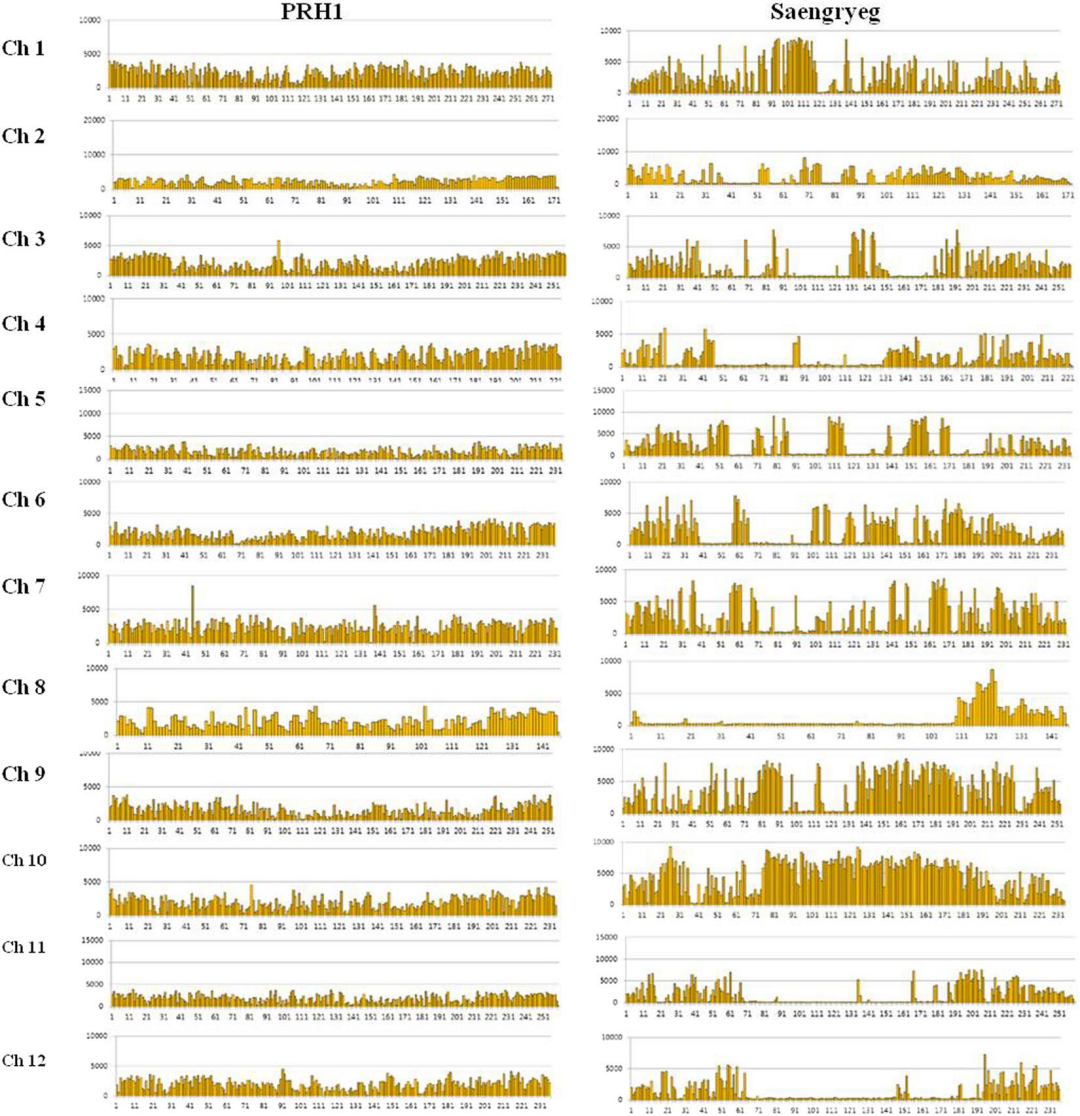
SNP distribution observed per 1 Mb chromosome. Thedistribution of SNPs detected with resequencing of pepper varieties along 12 chromosomes. The horizontalx-axis denotes the length (Mb) of chromosome and y-axis represents number of SNPs254x190 mm.

Genome-wide SNPs were identified using an improved BWA-SAMtools workflow. The high-quality filtered reads of PRH1 and Saengryeg were mapped to the reference genome. A total of 6,213,009 and 6,840,889 SNPs were identified for both pepper varieties. Based on the SNP ratio to the read map, SNPs were classified into homozygous, heterozygous and other types. Among the identified SNPs, 88.59% homozygous, 3.65% heterozygous, and 7.76% other types of SNPs were determined in PRH1. Likewise, in Saengryeg, 95.04% homozygous, 1.91% heterozygous, and 3.05% other type SNPs were identified. The occurrence of low percentage of heterozygous SNPs in both lines was due to the relatively low sequence depth and rigid SNP calling requirement. Capsicum consists of 12 chromosomes, and the SNPs are distributed evenly across all chromosomes. Our further analysis revealed that the number of SNPs differed in chromosome 1 to 12 for the two pepper varieties (Fig. 1). The greatest number of homozygous SNPs were noted in chromosome 10 (1,096,754) in Saengryeg whereas chromosome 1 consisted of maximum number of homozygous SNPs (601,032) in PRH1. Similarly, chromosome 1 in PRH1 possessed higher number of heterozygous SNPs (23,932) and chromosome 12 consisted of maximum heretozygous SNPs (15,942) in Saengryeg. However, the least number of SNPs was discovered on chromosome 8 in both pepper varieties. The detailed dataset for the chromosomal distribution of SNPs is listed in Table 2.

**Table 2:** Distribution of SNPs in the chromosomes of PRH1 and Saengryeg.

Chromosome No.	PRH1		Saengryeg	
	Homozygous	Heterozygous	Homozygous	Heterozygous
1	601,032	23,932	692,326	9,977
2	400,513	15,849	388,770	8,428
3	557,185	18,211	466,439	11,441
4	420,713	20,654	296,183	9,345
5	405,744	21,116	577,304	12,761
6	448,886	20,823	516,710	10,563
7	552,120	17,025	620,306	14,005
8	304,395	11,915	153,518	7,229
9	384,009	16,739	890,135	9,787
10	460,912	23,517	1,096,754	10,236
11	499,176	14,391	467,193	10,278
12	469,523	22,456	301,556	15,942
Total	5,504,208	226,628	6,467,194	129,992

### Annotation of SNPs based on their position in the pepper genome

**Figure 2:**
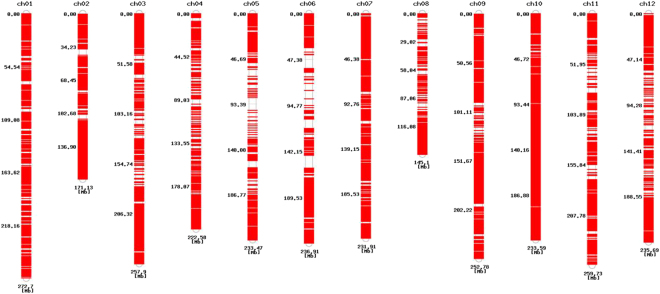
Genomic distribution of polymorphic SNP markers (PRH1 Vs Saengryeg) 254 × 190mm.

The SNPs were classified into two main categories (intergenic or genic region) according to their position in the pepper genome sequence. Further genic SNPs were sub-classified as intron and coding DNA sequences (CDS). A total of 6,213,009 and 6,804,889 genome-wide SNPs were discovered for PRH1 and Saengryeg, respectively. Of the discovered SNPs, 5,781,951 (93.06%) and 6,695,385 (93.39%) of intergenic SNPs were recognized for PRH1 and Saengryeg, respectively. Further, these SNPs were classified into homozygous, heterozygous and other type depending upon the ratio to read map. In addition, 82.28% and 93.58% of homozygous type SNPs were identified in the intergenic region for PRH1 and Saengryeg, respectively. We discovered that the number of SNPs in intron was greater than that of CDSs in the genic regions. Most of the SNPs were located in the intergenic regions and were classified as homozygous type (Table 3). All the identified SNPs were analyzed for polymorphisms between PRH1 and Saengryeg. A total number of 15,941,182 SNP loci were identified with respect to the reference genome. Of the identified SNP loci, 4,887,031 polymorphic and 469,978 non-polymorphic loci were identified between PRH1 and Saengryeg. The genomic distribution of polymorphic SNP markers is presented in Fig. 2. High-resolution melting (HRM) marker primers were identified by targeting SNPs to discriminate between two lines. Among the polymorphic SNPs, 4,164,456 HRM candidates were identified, and 597,434 primer sets were selected. A total of 306,871 HRM primer markers were recommended for further breeding purposes (Supplementary file S1). These sets of HRM primers possibly discriminate between the two lines.

**Table 3:** Summary of SNP classification by genome structure.

Sample	Total no. of SNP	Region	Total	Homozygous	Heterozygous	Other
PRH1	6,213,009	Introns	280,076	258,904	7,824	13,348
		CDS	150,932	133,436	7,025	10,471
		Genic region	431,058	392,383	14,851	23,824
		Intergenic region	5,781,951	5,111,825	211,777	458,349
Saengryeg	6,804,889	Introns	69,542	64,994	1,938	2,610
		CDS	39,955	34,212	2,449	3,294
		Genic region	109,504	99,210	4,388	5,906
		Intergenic region	6,695,385	6,367,984	125,604	201,797

### Chromosome-wise characterization of polymorphic SNPs

**Figure 3:**
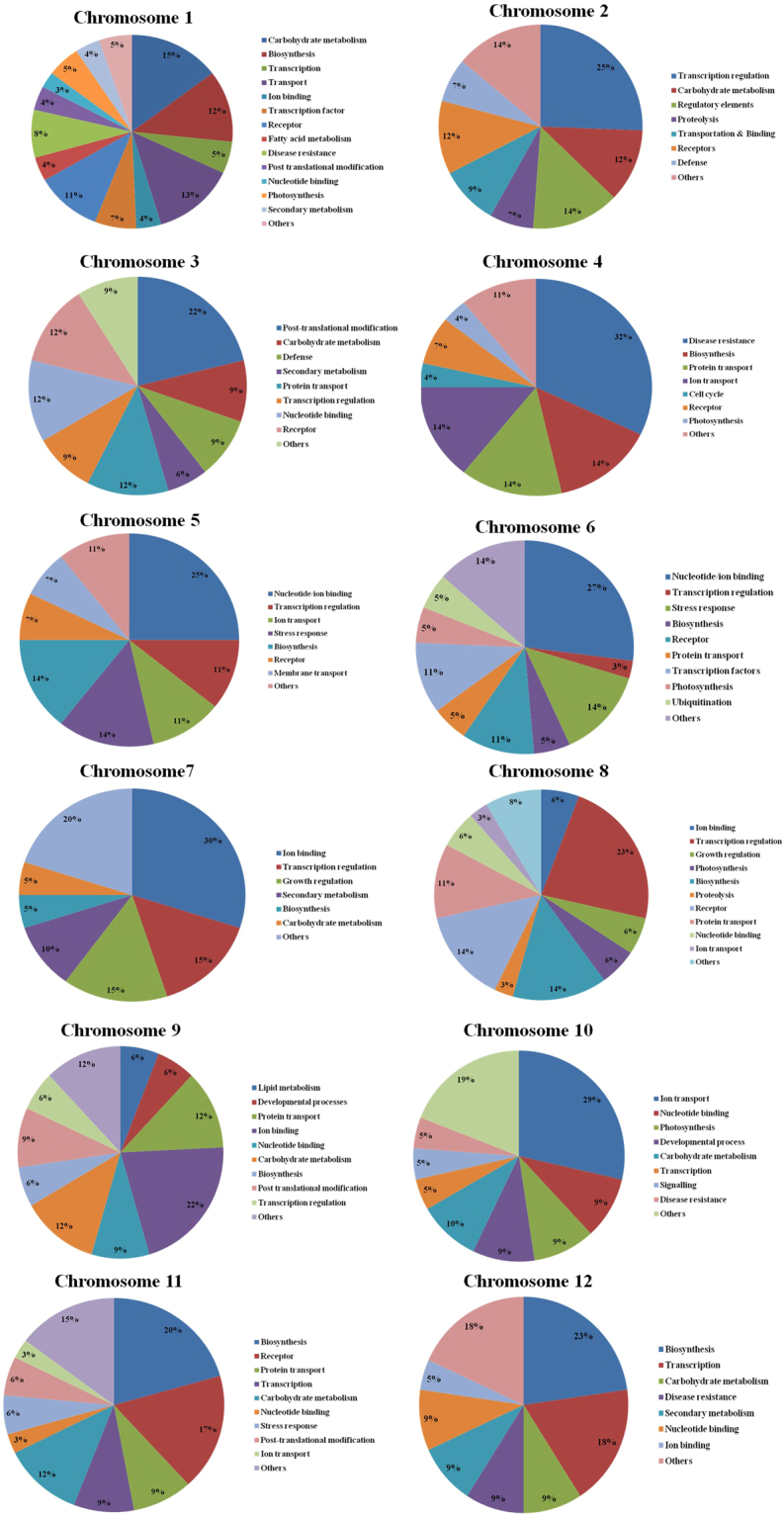
Chromosome wise annotation of polymorphic genic SNPs associated with important functionsinPRH1 and Saengryeg.

In order to gain deeper insight into the SNPs associated with the genes involved in disease resistance and stress tolerance process, chromosome-wide functional annotation of polymorphic SNPs were performed. The distribution of SNP markers were analyzed in each chromosomes and the functional characterization of genes with higher polymorphic SNPs have been carried out. Overall, the majority of the genes with high polymorphic SNPs widely involved in carbohydrate metabolism, transcription regulation, ion binding, nucleotide binding, protein transport, fatty acid metabolism, receptors, photosynthesis, post-translational modifications, stress response, regulatory elements, proteolysis, secondary metabolism, biosynthesis, diseases resistance, and others. However, in each chromosome the genes with various functions displayed the major proportion (Fig. 3). For instance, in chromosome 1 the SNPs were highly identified in genes involved in carbohydrate metabolism followed by transport related genes. Transcription regulation related genes consisted of numerous polymorphic SNPs in chromosome 2 and 8. In chromosome 3, the genes associated with post-translational modifications consisted of more polymorphic SNPs. Likewise the diseases resistances genes with high polymorphic SNPs dominated the chromosome 4. Moreover, nucleotide/ion binding and ion transport genes with polymorphic SNPs were identified in chromosomes 5, 6, 7, 9, and 10. Genes involved in biosynthesis consisted of vast number of SNPs in chromosome 11 and 12.

### Identification of polymorphic SNP markers associated with pathogen resistance genes

**Figure 4:**
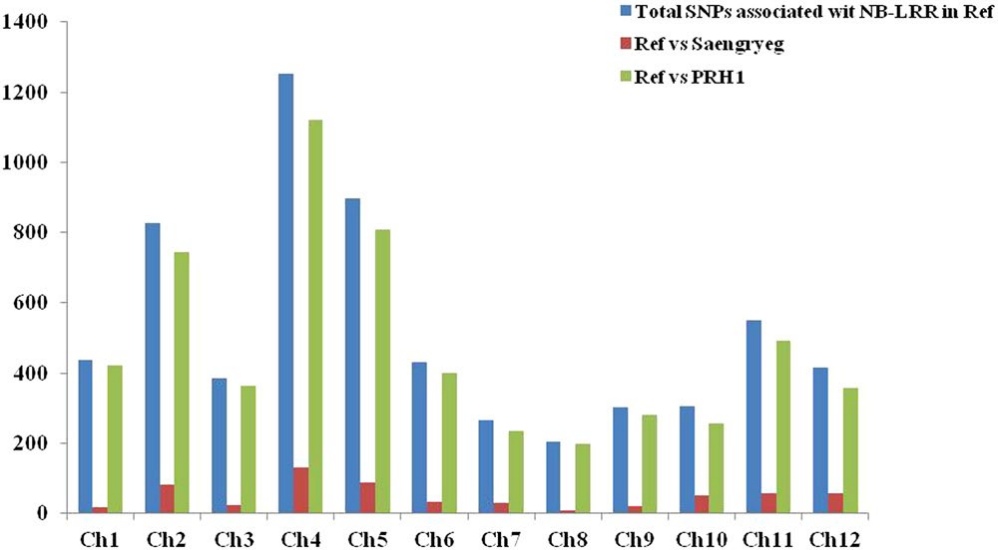
Chromosome wise occurrence of SNPs associated with NBS-LRR genesinPRH1 and Saengryegincomparison with reference genome.

In total, 6281 SNPs associated with 46 pathogen resistance genes with nucleotide binding site-leucine rich repeat (NBS-LRR) motif were identified in the introns and coding regions of the genes (Supplementary file S2). The occurrence of SNPs related to NBS-LRR genes in each chromosome has been listed in Fig. 4. The maximum number of SNPs was distributed in chromosome 4, whereas the least number of SNPs was observed in chromosome 8. Moreover, the PM resistant line PRH1 consisted of greater number of NB-LRR linked SNPs in comparison with the susceptible line Saengryeg. Overall, the occurrence of higher number SNPs particularly associated with the NB-LRR resistance genes could play a vital role in the attribution of PM resistance.

### Phenotypic evaluation for PM resistance and validation of SNP markers

**Figure 5:**
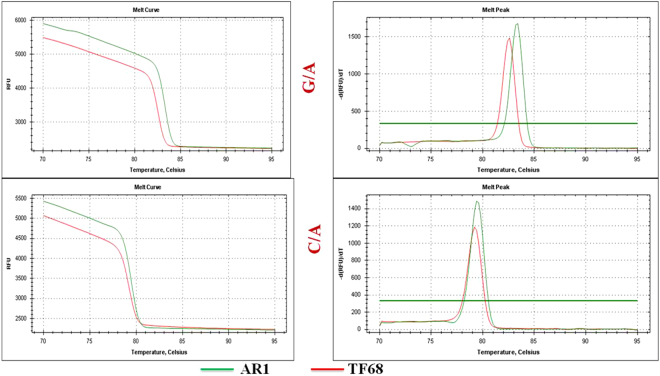
HRM melt curve and temperature peaks obtained from candidate SNPs between *C. baccatum* (AR1) and *C. annum* (TF68) illustrating the G/A and C/A SNP variation.

In order to assess the disease resistance indexes, the parental types and the F_4_ population were co-cultivated with the powdery mildew pathogen. The infection range observed in the plants has been categorized from 1–5 scale from PM resistance to susceptible (Supplementary Table file 3). The parents of F_4_ population exhibited contrasting degree of resistance to the PM disease. The *C. baccatum* variety (PRH1) displayed high resistance scale of 1, whereas the *C. annuum* variety (Saengryeg) exhibited resistance score of 5. However, among the 45 individuals in F_4_ population, 11 exhibited the resistance score of 1 followed by 22 plants resulted in the moderate disease resistance level of 3 and 12 plants displayed the severity with the index of 5. Further, to validate the identified SNP markers, HRM assay in both the parental types along with the F_4_ population of 45 progenies has been performed. Among the 36 HRM primers employed, 19 primers significantly distinguished the resistant and susceptible progenies in the F_4_ population. The HRM primers employed in this study have been listed in the Table 4.The representative HRM melt curves obtained for the parents with the heterozygous SNP variation of G/A and C/A have been illustrated in Fig. 5. Moreover the majority of heterozygous SNPs were observed to be prominent among the population studied. Thus, the current HRM platform provided a suitable approach for the validation of SNP markers among the population.

**Table 4:** List of HRM primers designed for genotyping polymorphic genic SNPs from each chromosome.

**Primer no**.	**Locus name**	**Gene name**	**5′-3′primer sequence**	**3′-5′primer sequence**	**Result of HRM analysis validated in F**_**4**_ **population**
1	CA01g00370	Serine/threonine protein kinase%2C putative	CGGCCAATGTATCAAGACTCG	AACGAATTCAACAACCGCGT	Positive
2	CA01g02310	Xpa-binding protein%2C putative	TCCCTTCTGCGGTTTTCCTC	TGTTGCAAACTTCTCCTTGTAGG	Positive
3	CA01g04020	Kinesin heavy chain%2C putative	CCCACTGGTGAAAGCAGTGT	TGGAGAGAAGGCCTCAATGG	Positive
4	CA02g00020	DNA-repair protein UVH3%2C putative	TGGTCAGGTAATGGTGGTTCT	CTCTCCCTCATCTGGCAAACA	Negative
5	CA02g00720	Pentatricopeptide repeat-containing protein%2C putative	AGAGCACTAACCTCTTTAGCA	GACTGCAAAGACCCCACAGA	Positive
6	CA02g02750	MYBR domain class transcription factor	ACAGTCATACTAGATGAAGGCGG	TGATGCAATGTGGTCAGATGA	Negative
7	CA03g00110	Beta-galactosidase	AGTAACTGATGGAATTTCGGAA	TGGATGCGTTTTAGCCTGACT	Positive
8	CA03g00740	Small subunit processome component-like protein	TCCCAGCATACTCGTCCAAC	CCTCAACCTAGGCATGCCAA	Negative
9	CA03g15330	PREDICTED: Golgi to ER traffic protein 4 homolog	TGGTTAGTCTTTCCTAATCCGGT	CTATTTCTTTTTCCATTCCATTGC	Positive
10	CA04g00830	Phosphatidylinositol 4-kinase%2C putative	GGGGGCTAGTCTTCTCTTCT	GGCAACAAGGTGGAAAGACG	Negative
11	CA04g00250	PREDICTED: transmembrane emp24 domain-containing protein p24beta3-like	CGGATCATCCCGGCATTGAT	TCACCTCCGATTCACAACTCA	Negative
12	CA04g00360	Protein transport protein sec. 23%2C putative	GCACGCCCATACCTTGTCAA	ATCAATGCCAAGCCCATCCA	Positive
13	CA05g00010	RNA polymerase II transcription mediators isoform 1	CAACGAGGCTGACCGAAAGA	CTCCACTCGCCCATCTTCTC	Positive
14	CA05g00320	Folylpolyglutamate synthase	GGTGGGGGCTTTTGTCTTCT	ACTACATCTTCTGAGGTAACACC	Negative
15	CA05g15050	PREDICTED: mediator of RNA polymerase II transcription subunit 33A	CCACCGTTTCAATCCCTTGC	ACGTGTCAGGATTCATAAGCT	Positive
16	CA06g00010	Kinesin heavy chain%2C putative	TGAAGCCGCCTCGAATTTCT	AATGAGACTTCGAGGGGCAC	Negative
17	CA06g01280	Myosin XI%2C putative	ATAGACCCCGGCTCAGGAAT	GCAAAGGTAGCTCCACCACT	Positive
18	CA06g01570	PREDICTED: TBC1 domain family member	GGCAGGAAGATACAATAAATGTAC	AGCAGTATCGTGATTTCATTTGGT	Negative
19	CA07g03700	PREDICTED: synaptotagmin-5-like	AGTAAGGTCAAATGTGGAGCCA	AGAACGTTAATACTGGCCATCG	Negative
20	CA07g04200	Transducin family protein	TGCGAACTTAAGGAAAAAGAAGCA	GTAATGCTTGTCGGGAGCCT	Positive
21	CA07g12460	Formin	GGGATAACGCTCTTCCATATGGA	CATGTCTGACAGAGGGTGCA	Negative
22	CA08g00950	Transcription cofactor%2C putative	ACACTGAGATGCATGCACCA	TACCTGGTTTTGGCTGTGTT	Positive
23	CA08g08740	DNA-directed RNA polymerase	ACAACAGGGACATGATTTCATCA	ACACTAAACCCTTCTGTGCACA	Positive
24	CA08g09730	PREDICTED: protein ZINC INDUCED FACILITATOR-LIKE 1-like isoform X3	TGTGTGTCGAAGCAATTGAT	CTGTTGGAAGATTTGTCAATATCA	Positive
25	CA09g00140	O-linked n-acetylglucosamine transferase	CTGCACATAGAATTCTTGCCCA	TGGGATTGTTTCGTGCTTTT	Negative
26	CA09g01180	Vacuolar protein sorting-associated protein	TTGTCCTCCTCCTCAGATGA	ACCACCAGCAAGAACGTCAA	Negative
27	CA09g14940	Beta-amyrin synthase	TGGCACCATTTTTAAACAACA	ACAGTCAGAAGCACACTGTGA	Positive
28	CA10g01250	PREDICTED: heterogeneous nuclear ribonucleoprotein	TGATGAGCTCGGAGGAGTCA	AAGTGGCTGGGATTCAAGGG	Negative
29	CA10g01280	Protein binding protein%2C putative	GGGTGAGTTTCCTAAGAGGTCC	CAAATCACATGGCCAAACGC	Positive
30	CA10g07870	Amidase%2C putative	GCTGCAGCAATGTAATTGGA	CCTCTGACCATCATCGCTGA	Negative
31	CA11g11870	Xanthine dehydrogenase	ACCTTGACTGGTACACTTTTTCA	AGTGATGACGGACAATTGTGT	Positive
32	CA11g15420	Tubulin family protein	GGCCTCATAACACCGTGGAA	TTACCAGCAGCATTGATCGA	Negative
33	CA11g15430	ABA aldehyde oxidase	TTAATGGAGGCTTCAGAGAGA	GCTTGGGACTCTTGAAAGAAGC	Positive
34	CA12g01070	IsoleucyltRNA synthetase%2C putative	ACAACACCCATCGACTTCCC	TGCAGAGCCAGATTTCAGGT	Positive
35	CA12g02370	N-like protein	TGGTGTTTTTCCATTTGCCT	TCTCTAGAACGTAAGGGTATTCA	Negative
36	CA12g22510	PREDICTED: pleiotropic drug resistance protein	ACCGAGTCGAAAGAGGAAGC	AAGGGCAGAGTCGAGCTTTC	Negative

## Discussion

In general, a primary requisite of genotyping of all the individuals in a population is necessary for trait mapping in traditional approaches of breeding, which is a highly expensive, labor intensive and time consuming process. Moreover, the occurrence of mere levels of variations or polymorphism also acts as a vital challenge during molecular marker discovery. In order to address these difficulties, next generation sequencing (NGS) strategies have been widely applied in genomics based on breeding of important agricultural and horticultural crops. Recent advancements in NGS technology have facilitated the routine use of high-throughput, low-cost markers for plant breeding programs. New software tools enable the discovery, validation, and assessment of genetic markers on a large scale. Among different marker systems, SNPs are the most important and attractive DNA-based molecular markers used for genetic diversity and genome association analyses and comparative genetics in plant breeding^6–9^. SNP markers are highly polymorphic, co-dominant, precise, reproducible, high-throughput, economical and informative^28^. Moreover, the discovery of genome-wide SNPs aids in the improvement of marker assisted selection, particularly for the identification of traits associated with disease resistance. In this study, a complete genome resequencing of two pepper varieties with contrasting powdery mildew (PM) tolerance ability, PRH1 (PM resistance) and Saengryeg (PM susceptible), has been examined for the identification of SNP markers associated with powdery mildew resistance. The available whole genome sequence information of *Capsicum annuum* cv CM334 has been utilized as the reference genome to enable the comparison between the *C. annum* and *C. baccatum* lines used in this study.

In the current endeavor,intersepecific breeding of sexually incompatible pepper species has been performed due to their potential traits. For instance, the *C. baccatum* is well-known for fruit quality, disease resistance, and high contents of valuable secondary metabolites^29^. Therefore, the interspecific breeding of peppers results in progenies with high fruit quality and disease resistance. The *C. baccatum* variety used in this study displayed resistance to powdery mildew and anthracnose diseases. Hence, the whole genome re-sequencing (WGRS) based on discovery of SNPs in the variable pepper varieties could enhance the understanding of SNPs associated with disease resistance. The resequencing and SNP discovery resulted in the identification of 6,213,009 SNPs for PRH1 and 6,840,889 SNPs for Saengryeg. The SNPs identified in the present study were higher than the SNPs discovered by Nimmakayala *et al*. in *C.annuum* and *C.baccatum* varieties using genotyping by sequencing approach^30^. The report suggested the collective identification of 36,621 potential SNP markers linked to various genomic regions in in *C. annuum* and *C. baccatum* that can be utilized for the genome wide association studies in pepper varieties^30^. Moreover, the identified SNPs in the present study have been majorly categorized into homozygous type with 88.59% and 95.04% for PRH1 and Saengryeg, respectively. This suggests that the sequence of reference genome could be generated from homozygous loci. Further, the chromosomal distribution of SNPs in the pepper genome revealed that a total of 10.92% of homozygous SNPs were located on chromosome 1, and 16.96% of the homozygous SNPs were located on chromosome 10 for PRH1 and Saengryeg, respectively.

In addition, the distribution of SNPs within the pepper genome illustrated the occurrence of higher percentage of SNPs in intergenic regions compared with genic regions. Likewise, several SNPs were identified in the intronic region than in CDSs. Similar results were also reported in tomato by Kim *et al*.^31^. Furthermore, the location of SNPs plays a vital role, particularly SNPs should be located in intragenic regions to implicate the phenotypic traits. These SNPs are expected to be applied to marker assisted selection because they could be considered as functional markers. A total of 5,941,182 SNP loci have been detected between Saengryeg and PRH1. Of them, 30.63% SNPs were distributed in polymorphic loci. Potential polymorphic homozygous SNPs were filtered to discover breed-specific markers in both of the pepper varieties. HRM analysis has been applied to identify precise, cost-effective and efficient tool to detect sequence variations, such as SNPs^32^. This technique has been successfully implemented to identify SNPs that have been used for genotype discovery, genetic mapping and mutation scanning^33–36^. Among the discovered homozygous type polymorphic SNPs, 597,434 HRM marker primers were identified that potentially discriminate between two lines. Of them, 306,871 HRM primers were recommended for further experimental research related to PM-based melting patterns and amplification efficacy.

The numerous amount of polymorphic SNPs identified in the genic region were functionally annotated in each chromosome to gain deeper insight into the SNPs associated with the genes involved in disease resistance. A comparative genetics study on the resistance genes in Solanaceae family has shed light on to the potential loci in different chromosomes linked with disease resistance^37^. The vital R genes associated with disease resistance were conserved among the related species such as pepper, tomato, and potato^37^. The current results revealed that each chromosome consisted of several SNPs associated with the genes involved in vital metabolic processes. However, chromosome 4 consisted of larger set of SNPs associated with disease resistance in comparison with other chromosomes. According to Grube *et al*.^37^, the diseases resistance gene loci located in the chromosome 4 of pepper could render resistance against fungal pathogens. Correspondingly, chromosome 4 could play a vital role in encompassing the genes required for disease resistance in pepper. Moreover, the chromosome 5–10 consisted of SNPs related to genes involved in ion and metal binding. The roles of ion/metal binding genes are inevitable particularly under stressed conditions in pepper plants. The uptake and transportation of nutrients and water from the environment to the plant is a complex as well an important process for the improvement of physiological functioning of plants in stress. Hence, the SNPs related to these genes could act as a vital marker under stress.

Furthermore, higher number of polymorphic SNPs associated with disease resistance genes such as NBS-LRR were also identified in chromosome 4. Among the two varieties, the resistant PRH1 possessed higher distribution of polymorphic SNPs related to NBS-LRR genes. In plants, NBS-LRR is a large family of proteins encoded by the resistance genes and NBS-LRR proteins involved in the recognition of pathogens^38^. Several reports suggested the importance of NBS-LRR proteins in the resistance against numerous diseases including powdery mildew in plants^39–41^. In the present study, polymorphic SNPs were identified in genes encoding for LRR receptor-like serine/threonine-protein kinase, F-box/LRR, TIR-NBS-LRR resistances protein, CC-NBS-LRR resistance protein, and TIR1 like protein, etc. Hence, the identification of SNPs associated with the disease resistance genes could aid in the enhancement of screening processes in the molecular breeding of pepper with powdery mildew resistance.

The identified SNPs were validated using HRM primers in the parents and F_4_ population derived from the *C. annuum* and *C. baccatum* varieties. The HRM primers were selected from all the chromosomes and evaluated in the parents and the population. Among the tested primers, 19 primers were able to distinguish the population and the results were correlated with the phenotypic disease evaluation scores for each individual. Overall, the polymorphic SNPs discovered in this study can be utilized for the identification of powdery mildew resistance and susceptible cultivars in pepper breeding. However, in future the present investigation will be extended to evaluate large populations with more number of HRM primers corresponding to important SNPs associated with powdery mildew resistance in pepper.

In summary, the present endeavor reports the discovery of numerous SNP markers with potential applications in population genetics, molecular breeding, linkage mapping, and comparative genomics on gene-based association studies. For the first time, polymorphic SNPs were discovered from *C. annuum* and *C. baccatum* varieties of pepper with different powdery mildew resistance property. The SNP information obtained from the current WGRS approach in pepper can be utilized for the genomics assisted breeding of Capsicum with powdery mildew resistance.

## Methods

### Isolation of genomic DNA from pepper plants

Young leaves of PRH1 and Saengryeg were used for genomic DNA isolation. Briefly, 300 mg of leaves were ground into fine powder using liquid nitrogen. High-quality DNA was extracted using the cetyltrimethylammonium bromide (CTAB) extraction method^42^. Powdered samples were mixed with CTAB buffer and incubated at 65 °C for 10 minutes. Sample mixtures were cooled to room temperature, and chloroform was then added to the sample mixture. Chloroform sample mixtures were mixed thoroughly and centrifuged at 13,000 rpm for 5 minutes at 4 °C. The supernatant was transferred into a new tube, and an equal volume of absolute ethanol was added. The solution was centrifuged at 13,000 rpm for 5 minutes at 4 °C, and the supernatant was discarded. Then, 70% ethanol was added to the sample, which was then centrifuged at 13,000 rpm for 5 minutes at 4 °C. Once again, the supernatant was discarded, and precipitated DNA pellets were dried at room temperature. The precipitated DNA pellets were then used as a starting material for purification using the Sigma Genelute plant DNA isolation kit (G2N70, Sigma). The DNA quality was assessed by electrophoresing the DNA on 1% agarose gel. The concentration of the extracted DNA was estimated using a GE Healthcare Bio-Science NanoVue via assessment of a single absorbance peak at 260 nm, a 260/280 absorbance ratio of 1.8 to 2.0 and no evidence of substantial band shearing or contamination (either RNA or polysaccharide).

### DNA library construction and massively parallel sequencing

Purified whole genomic DNA was randomly sheared using a Covaris S2 (Covaris, Woburn, MA) to yield DNA fragments in the target range of 400 to 500 bp, and average molecular sizes were assessed using an Agilent Bioanalyzer 2100 (Agilent Technologies, Palo Alto, CA). Subsequently, the resulted overhangs were converted to blunt ends using a TruSeq DNA Sample Preparation Kit v2 (Illumina, CA, USA) followed by a clean-up protocol using AMPure XP Beads (Beckman Coulter Genomics, Danvers, MA). To enhance the ligation between the fragmented DNA and index adapters and to avoid self-ligation, the 3′ ends were adenylated. After adenylation, the index adapters were ligated to the fragmented genomic DNA, and the ligated products were purified using the AMPure XP Beads. The ligated products were size-selected on a 2% agarose gel followed by gel elution and column purification. The selected ligated DNA fragments with adapter sequences were enhanced through PCR using adapter-specific primers. Further, the DNA was re-isolated and the average molecular sizes of the libraries were evaluated using the Agilent Bioanalyzer 2100 (Agilent Technologies, Palo Alto, CA) to assess a sharp peak in the expected 500–600 bp range. Each library was loaded on the HiSeq. 4000 platform, and the high-throughput sequencing was performed to ensure that each sample met the 10-fold average sequencing depth.

### Preprocessing

After sequencing, the raw reads were trimmed using the Solexa QA v.1.13 package (Cox *et al*., 2010). The quality of bases from either end of Illumina reads commonly drop in, therefore either end of the reads were trimmed when the Phred quality score dropped below Q = 20 (or 0.05 probability of error). In addition, all 5′ and 3′ stretches of ambiguous ‘N’ nucleotides were also clipped. Trimming resulted in reads with a mean length of 101 bp across all samples, and a minimum length of 25 bp was applied during sequence trimming. These data were used for downstream analysis. The reference genome sequence of *Capsicum annuum* cv. CN334 was downloaded from Sol Genomic Network (SGN) at http://www.sgn.cornell.edu/.

### Alignment, detection, and annotation of SNPs

To align the reads to the pepper reference genome, the Burrows-Wheeler Aligner (BWA 0.6.1-r104) program^43^ was applied. The BWA default values for mapping were used, except for seed length (−l) = 30, maximum differences in the seed (−k) = 1, number of threads (−t) = 16, maximum number of gap extensions (−e) = 50, mismatch penalty (−M) = 6, gap open penalty (−O) = 15, and gap extension penalty (−E) = 8. Mapped reads were extracted from the resulting BAM file using SAMtools 0.1.16^44^ for further analyses. The high mapping quality ensures reliable (unique) mapping of the reads, which is important for variant calling. Using the varFilter command, SNPs were called only for variable positions with a minimal mapping quality (−Q) of 30. The minimum and maximum of read depths were set as 3 and 100, respectively. An *in-house* script considering biallelic loci was used to select significant sites in the called SNP positions^31^. Depending on the ratio of SNP reads to mapped reads, variant types were classified into three categories: homozygous SNP (more than 90%), heterozygous SNP (morethan 40% and less than 60%), and other SNPs for the remaining types. The polymorphic SNPs between two samples with sufficient sequences on both sides of the SNP site, without structural variation were noted adjacent to the SNP site and selected for primer design. To design primers flanking the SNP, an *in-house* script and Primer3 (v2.3.5) software were used^45^. The parameters employed for the primer designing areas follows, primer length 18–24 bp, with 20 bp as the optimum; primer GC% = 20–80%, with the optimum value being 50%; primer Tm 55–65 °C, with 60 °C as the optimum; and product size range of 80–600 bp. After the designed primers were mapped to the genome sequence, only the primers that aligned were selected as candidates for SNP markers.

### Functional annotation of genic SNPs

The functional annotations of polymorphic SNPs were determined using the information acquired from gene ontology consortium (www.geneontology.org) and Gene Ontology (UniProt) (www.uniprot.org/help/gene_ontology). The number of SNPs associated with each gene was identified manually.

### Genotyping of SNPs using high resolution melt assay (HRM)

For the SNP validation, HRM primers were designed from each chromosome and evaluated in 46 F_4_individuals and compared with the parental lines. The HRM analyses were performed in 20 μl of total reaction mixture containing 2 μl of DNA extract (200 ng), 1× of SsoFastEvagreenSupermix (Bio-Rad Laboratories, Hercules, CA, USA), and 200 nM of forward and reverse primers. The reactions were performed in a fluorometric thermal cycler CFX96 real-time system (Bio-Rad Laboratories, Hercules, CA, USA), following program: 98 °C for 2 min, 45 cycles at 98 °C for 5 s and 60 °C for 10 s. The peaks obtained were normalized and analyzed for the difference in the melt curve.

### Physiological disease resistance evaluation

The HRM results were correlated with the physiological evaluation of disease resistance. For the infection of powdery mildew, the parental lines used in this study, *C. annuum* - TF68 and *C. baccatum* – ARI are the close relatives of PRH1 and Saengryeg. The parents as well as the F_4_ populations were maintained in a polyvinyl house along with disease infected plants under a normal day light condition with night/day set temperatures of 27/15 °C and 60–70% RH. The experiment was performed in triplicates in random block design. The disease severity has been assessed in 1–5 scale (1-resistant, 3-moderate and 5- sensitive) after two weeks.

## Electronic supplementary material

Supplementary Information.

Supplementary Dataset 2

Supplementary Dataset 1
