# Supplementary material for: Whole Genome Resequencing of Capsicum baccatum and Capsicum annuum to Discover Single Nucleotide Polymorphism Related to Powdery Mildew Resistance
Source: Sci Rep. 2018 Mar 26;8:5188. doi: 10.1038/s41598-018-23279-5 (PMC5980001; doi:10.1038/s41598-018-23279-5)
Supplement: Supplementary file 1 — Supplementary Information. [file 41598_2018_23279_MOESM1_ESM.pdf]

Whole Genome Resequencing of *Capsicum baccatum* and *Capsicum annuum* to Discover Single Nucleotide Polymorphism Related to Powdery Mildew Resistance

Yul-Kyun Ahn[1*], Abinaya Manivannan[1], Sandeep Karna[1], Tae-Hwan Jun[2], Eun-Young Yang[1], Sena Choi[1], Jin-Hee Kim[1], Do-Sun Kim[1], and Eun-Su Lee[1]

Supplementary Table 3. Phenotypic evaluation of F_4 population for powdery mildew resistance.

**Parent / Population (AR1xTF68)**	**Disease score**	**Inference**
AR1	1	Resistant
TF68	5	Sensitive
1	5	Sensitive
2	5	Sensitive
3	3	Moderate
4	3	Moderate
5	5	Sensitive
6	3	Moderate
7	5	Sensitive
8	3	Moderate
9	3	Moderate
10	3	Moderate
11	3	Moderate
12	5	Sensitive
13	3	Moderate
14	5	Sensitive
15	3	Moderate
16	3	Moderate
17	3	Moderate
18	5	Sensitive
19	5	Sensitive
20	3	Moderate
21	1	Resistant
22	3	Moderate
23	3	Moderate
24	5	Sensitive
25	5	Sensitive
26	3	Moderate
27	1	Resistant
28	1	Resistant
29	3	Moderate
30	3	Moderate
31	3	Moderate
32	5	Sensitive
33	1	Resistant
34	1	Resistant
35	1	Resistant
36	3	Moderate
37	3	Moderate
38	3	Moderate
39	5	Sensitive
40	1	Resistant

41	1	Resistant
42	1	Resistant
43	1	Resistant
44	1	Resistant
45	3	Moderate
